# A Hypothesis for the Possible Role of Zinc in the Immunological Pathways Related to COVID-19 Infection

**DOI:** 10.3389/fimmu.2020.01736

**Published:** 2020-07-10

**Authors:** Ander Mayor-Ibarguren, Carmen Busca-Arenzana, Ángel Robles-Marhuenda

**Affiliations:** ^1^Department of Dermatology, La Paz University Hospital, Madrid, Spain; ^2^Department of Internal Medicine, La Paz University Hospital, Madrid, Spain

**Keywords:** COVID-19, SARS-CoV-2, treatment, zinc, IL-6

## Key Points

- Zinc deficiency may be common and associated with severe infection.
- Zinc helps to enhance the interferon type 1 response to the virus and participates in many regulatory pathways.
- Low levels of zinc have been associated with higher IL-6 responses.
- IL-6 plays an important role in severe lung injury due to COVID-19 infection.
- Zinc inhibits SARS-CoV RNA polymerase, and thus its replication capacity.
- Zinc may increase the efficacy of antimalarial agents, since they are zinc ionophores.
- Differences in mortality due to COVID-19 infection may be explained to some degree by−174 IL-6 gene polymorphism.

Zinc (Zn) is the second most abundant trace metal in the human body after iron. However, unlike iron, there is no specialized zinc store (1). Zinc's functions can be classified as catalytic, structural, and regulatory (2). For example, important zinc metalloenzymes include alkaline phosphatase, RNA polymerases, and alcohol dehydrogenase (3). Zinc deficiency can precipitate an immune system imbalance, exemplified in severe deficiency by high susceptibility to infections, skin disorders, gastrointestinal disorders, weight loss, growth retardation and male hypogonadism, amongst other symptoms (4). While severe zinc deficiency is rare, mild to moderate deficiency is more common worldwide (5). There are very low levels of free zinc in plasma, since it is mostly bound to proteins such as albumin, alpha-2-macroglobulin (A2M), and transferrin. Plasma zinc levels are therefore only around 1 μg/ml, equal to 0.1% of total body zinc, but are still the most important reservoir for zinc homeostasis, which requires “free” or “labile” zinc mobilization (6, 7). Kinetic studies suggest that only a small proportion of total body zinc (10%) represents the “functional pool” of zinc, located within the liver and other tissues, that exchanges rapidly with that found in the plasma (8, 9). When this functional pool is depleted, zinc deficiency ensues (8). Intracellular zinc is distributed in zinc-storing vesicles called zincosomes, the nucleus and other organelles. In cytoplasm, zinc mostly binds zinc-chelating proteins called metallothioneins (MTs). Zinc homeostasis is understood to be the correct balance of zinc distribution. Internal zinc homeostasis is regulated by the cooperative activities of two metal transporter protein families. One family consists of 10 solute-linked carrier 30 (SLC30 or ZnT) exporters, and the other family consists of 14 solute-linked carrier 39 (SLC39 or ZIP) importers (10, 11). For instance, most labile zinc in the body is absorbed by intestinal epithelial cells via SLC39a4 protein, and excessive zinc is excreted through the kidneys, and the intestine via SLC39a5 (12).

**Figure 1:**
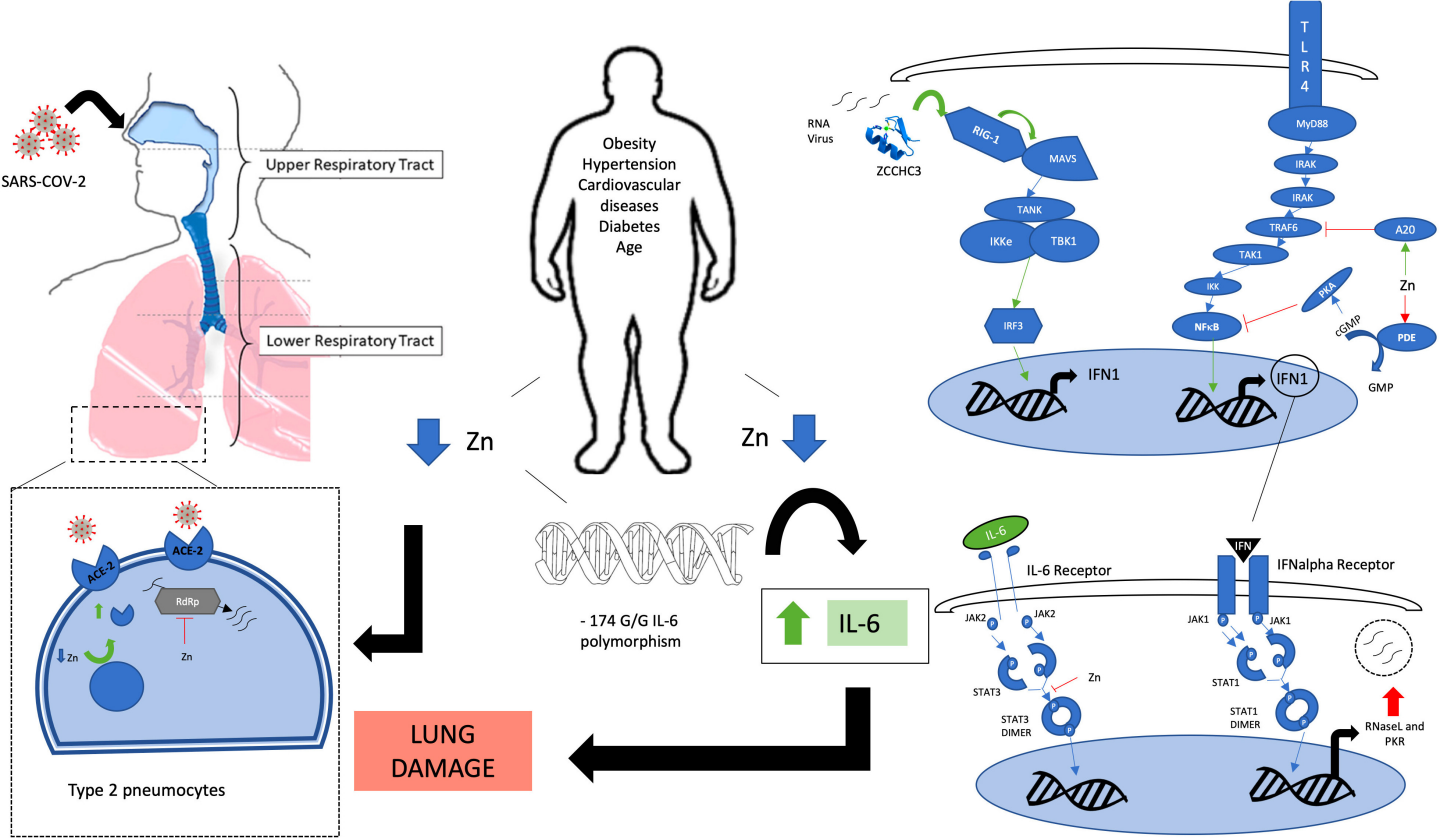
Legend: A schematic view of the involvement of zinc in various signaling pathways. Green arrows: zinc-mediated activation. Red T bar arrows: zinc-mediated inhibition. Blue arrows: Flow of activation pathway. Obesity, cardiovascular disease, diabetes, and aging are associated with zinc deficiency. −174 GG polymorphism on the IL-6 promoter gene is associated with zinc homeostasis impairment and elevated IL-6 levels which contribute to lung damage. Zinc deficiency may increase ACE-2 receptor activity on type 2 pneumocytes and other cells that are infected by SARS-COV-2, mainly in the lower respiratory tract. Zinc inhibits RdRP, blocking viral RNA replication. Zinc-finger protein ZCCHC3 senses viral RNA and activates through RIG-1-like receptor a cascade that results in an increase in the interferon type 1 response. IFN type 1 stimulates synthesis of antiviral proteins such as RNaseL and PKR. Zinc helps to regulate the same kind of responses by activating the A20 protein that inhibits TRAF6 downstream activation, and by inhibiting PDE, which results in increased levels of cGMP that will activate PKA that will inhibit NF-κB. Zinc also inhibits STAT-3 dimerization, blocking active STAT3 signaling from the IL-6 receptor. Acronyms: ZCCHC3: Zinc finger CCHC domain-containing protein 3. RIG-1, retinoic acid-inducible gene I; MAVS, Mitochondrial antiviral-signaling protein; TANK, TRAF family member-associated NF-κB activator; Iκkε, I kappa B kinase epsilon; TBK1, TANK binding kinase 1; IRF3, interferon regulatory factor 3; TLR, Toll-like receptor; MyD88, myeloid differentiation primary-response protein 88; IRAK, interleukin-1 receptor-associated kinase; TRAF-6, tumor necrosis factor receptor-associated factor 6; TAK1: IKK, I kappa B kinase; NFκB, nuclear factor kappa B; A20, zinc protein; PDE, phosphodiesterase; cGMP, cyclic guanosine-monophosphate; GMP, guanosine-monophosphate; PKA, protein kinase A; INF-1, interferon type 1; JAK, janus kinase; STAT, signal transducer and activator of transcription; RNase L, Ribonuclease L; PKR, RNA-activated protein kinase; ACE-2, angiotensin-converting enzyme 2; RdRp, RNA-dependent RNA polymerase.

We are currently experiencing an unprecedented COVID-19 pandemic caused by a novel RNA coronavirus called SARS-CoV-2, which can produce a severe acute respiratory distress syndrome (ARDS) (13). It was first detected in Wuhan province in China at the end of 2019 (14), and on 11 March 2020, WHO characterized COVID-19 as a pandemic (15). The reported mortality rate for those infected varies between countries (0.5–7.7%) with the most important focus previously in Italy and Spain and currently in the USA, UK, and Brazil (16–19). Age, male sex, and pre-existing chronic metabolic diseases including diabetes, cardiovascular disease, and obesity are associated with greater severity of infection (20). There is no specific treatment yet. Many agents are being used with variable success, but none have had their efficacy demonstrated in clinical trials. Examples include: antimalarial agents such as chloroquine and hydroxychloroquine, antivirals such as lopinavir/ritonavir and remdesivir, and tocilizumab as an anti-interleukin 6 (IL-6) receptor antibody (21–24). Remdesivir has shown good initial clinical outcomes in a clinical trial, when treatment started within 10 days of symptom onset (25). In contrast, in a double-blind randomized trial of 237 patients with severe COVID-19 (hypoxia and radiographically confirmed pneumonia) in China, time to clinical improvement was not statistically different with remdesivir compared with placebo taken for 10 days (median time to improvement 21 vs. 23 days; hazard ratio for improvement 1.23 [95% CI 0.87–1.75]) (26). Mixed and controversial results have also been published regarding antimalarial agents (27). Thus, more robust data are needed before conclusions can be drawn regarding treatment. IL-1 and IL-6 may play an important role in severe lung inflammation, leading to acute respiratory distress syndrome, which can result in patient death (28, 29). This pathway appeared relevant in SARS-CoV, producing severe acute respiratory syndrome (SARS), and in MERS-CoV, producing Middle East respiratory syndrome (MERS) (30). High serum levels of pro-inflammatory cytokines [IL-1, IL-6, IL-12, interferon γ (IFN-γ), and transforming growth factor-β] and chemokines (CCL2, CXCL9, CXCL10, and IL-8) were found in patients with SARS with severe disease compared with individuals with uncomplicated SARS (31). MERS-CoV infections of dendritic cells and macrophages result in robust and sustained production of pro-inflammatory cytokines and chemokines such as TNF-α, IL-6, CXCL-10, CCL-2, CCL-3, CCL-5, and IL-8 (32). The purpose of this article is to highlight the key roles that zinc can play in COVID-19 infection (summarized in Figure 1), based on pre-existing evidence of its role in immune system function and viral infections, as well as its estimated possible deficiency in at-risk populations.

Zinc deficiency may be present in up to 17% of the population worldwide. The elderly especially are at higher risk of zinc deficiency and its adverse effects (33). Impairment of zinc homeostasis has also been demonstrated in metabolic diseases including diabetes, obesity, and cardiovascular disease (34). Many antihypertensive drugs such as ACE inhibitors, angiotensin 2 receptor antagonists, and thiazide diuretics are zinc chelators (35). Iron and calcium may interfere with zinc absorption too (36). The US Food and Nutrition Board recommends intake of 11 and 8 mg/day for adult men and women, respectively (37). Apart from calcium and iron, non-digestible plant ligands such as phytate, some dietary fibers, and lignin chelate zinc and inhibit its absorption. Measurement of plasma zinc levels is the most useful clinical test for zinc deficiency, despite limited sensitivity, and specificity (38). Also, plasma zinc levels remain stable even with low dietary intake, due to homeostasis in the body, decreasing in blood only when deficiency is very prolonged (39). The absence of a dedicated store for zinc repletion results in impairment of function when zinc status is compromised. Homeostasis maintains a constant intracellular zinc concentration and a plasma concentration within the reference range of 11–25 μM (0.7–1.6 mg/L) (40). Low plasma zinc has been defined as <60 mcg/dL (<9.2 μM) (41). When zinc intake decreases, homeostatic mechanisms initially maintain the plasma concentration within the reference range, but when deficiency is severe or prolonged, the concentration decreases. However, although plasma zinc concentration moderately correlates to habitual intake, the test also has limited specificity because zinc levels are depressed during inflammatory disease states or pregnancy and increase with acute catabolic states (42). In mild diseases, with C-reactive protein (CRP) levels of 15 mg/L, a 10% decrease in zinc is observed. In severe infectious diseases, CRP levels can reach 100–200 mg/L, with a much greater decrease in zinc levels (40–60%) (43). If CRP levels are normal, plasma zinc measurements are more reliable. Moreover, the test has limited sensitivity since patients with mild zinc deficiency may have normal plasma levels (44). The copper:zinc ratio may be an interesting marker for the diagnosis of zinc deficiency, since the latter leads to an increase in copper absorption (45). To be reliable, this ratio must be higher than 1.5. However, critical patients may have high levels of copper, reflecting the effects of the systemic inflammatory response, thus not reliably representing their actual levels (46). A marker that might be more sensitive to the nutritional status of zinc is the ratio of Apo/Holo activities of angiotensin converting enzyme (47). Zinc levels may also be measured in neutrophils, lymphocytes, or erythrocytes, but these assays generally have poor sensitivity (48, 49). Ruz M et al. reported that zinc levels in neutrophils do not change, even in the event of changes in plasma concentrations during experimentally-controlled zinc depletion (48). Metfah et al. found that zinc levels in lymphocytes, granulocytes, and platelets decreased significantly only during the late zinc depletion phase (49). Interestingly, plasma zinc levels did not change even during the late zinc depletion phase in this study. In contrast, they found that activity of ecto-5′-nucleotidase (an integral zinc-dependent plasma enzyme located on most mammalian cells) was significantly decreased during mild zinc deficiency. When measured in neutrophils, zinc deficiency is defined as <42 mcg/10^10^ cells (49). When measured in lymphocytes, zinc deficiency is defined as <50 mcg/10^10^ cells (49). Taking all this into account, we highlight that there is no good reliable definition for zinc deficiency, besides a low plasma concentration with respect to normal reference levels, which may not be representative, especially in acute states or mild grades.

Zinc homeostasis in immune system pathways is complex, since it participates both in pro-inflammatory and regulatory pathways, and much of the data comes from preclinical *in vitro* studies. Despite this, it seems clear that deficient or excessive zinc levels can lead to malfunction of the adaptive and innate immune systems. Zinc regulates the proliferation, differentiation, maturation and functioning of lymphocytes, and other leukocytes (6). It also regulates the immune response, and its deficiency increases susceptibility to inflammatory and infectious diseases, including pneumonia (50). Zinc sulfate supplementation at 20 mg/day for 5 months reduced acute lower respiratory tract infection morbidity vs. placebo in a clinical trial (51). Zinc is essential in both the adaptive and innate immune systems (52). For instance, the functionality of natural killer (NK) cells, which are essential for maintaining the immune response against viruses and tumors, is affected by low levels of zinc (53). Furthermore, zinc supplementation significantly increased NK cell numbers in whole blood cultures and NK cell activity *in vivo* (54, 55). In this latter study, zinc supplementation in subjects with low or borderline-normal circulating zinc increased the concentration of this ion and improved NK lytic activity, as well as modulating plasma IL-6. Zinc homeostasis directly influences the formation of lymphocytes and the secretion of cytokines and indirectly alters their stimulation by the innate immune system (56). There is also evidence that unregulated zinc homeostasis in macrophages impairs phagocytosis and results in an abnormal inflammatory response (57). In a study performed in mice, a diet deficient in zinc was associated with more pronounced airway inflammation after agricultural organic dust exposure, compared with normal dietary zinc intake (58). This was partially explained by the fact that macrophages maintained in a zinc-deficient environment exhibited increased CXCL1 and Il-23 production, as a result of increased NF-kB activation. Also, pulmonary zinc deficiency may be one of the mechanisms by which HIV-1 infection impairs alveolar macrophage immune function and facilitates severe pulmonary infection in these individuals (59).

Zinc also has a role in viral recognition. The zinc-finger protein ZCCHC3 binds RNA and facilitates the detection of intracellular RNA viruses by activating retinoic acid-inducible gene-I (RIG-1)-like receptors (RLRs), including RIG-I and MDA5 (60). This action triggers the activation of the anti-viral response mediated by downstream activation of antiviral genes (61). In this process, kinases such as TBK1 and IκK further phosphorylate the interferon regulatory transcription factor 3 (IRF3) and IκB-alpha, the NK-κB inhibitor, leading to activation of IRF3 and NF-κB, which results in interferon type 1 upregulation (62, 63) (see Figure 1). Interferon alpha-induced signaling results in upregulation of antiviral proteins (RNase L and PKR), known to degrade viral RNA and inhibit its translation (64). Zinc also exerts an inhibitory effect on the activation of NF-κB, through the expression of the A20 protein. A20 is a zinc-finger protein that negatively regulates tumor necrosis factor receptor (TNFR) and toll-like receptor (TLR)-initiated NF-κB pathways (65). Furthermore, zinc acts as an inhibitor of cyclic nucleotide phosphodiesterase (PDE). When PDE is inhibited, cyclic nucleotide cGMP (cyclic guanosine monophosphate) is elevated, leading to the activation of PKA (protein kinase A), and subsequent inhibition of NF-κB (66). Additionally, zinc supplementation has been shown to downregulate inflammatory cytokines by decreasing gene expression of IL-1β, TNF-alpha, and by inhibiting NF-κB activation (67).

Nutritional immunity is a process by which the host organism sequesters trace minerals during an infection so that their availability to pathogens is limited (1). During infection and inflammation, there is a transient transfer of zinc from serum to the organs, causing temporarily low serum zinc levels, which normalize during resolution of the inflammatory response (6, 7). Thus, a sufficient level of zinc is essential during responses to infection. Zinc signals act in an anti-inflammatory manner during sepsis by regulating the pro-inflammatory response, due to cellular uptake of zinc by ZIP14 as shown in a polymicrobial model of sepsis in mice (68). Zinc deficiency was strongly associated with an elevated risk of exaggerated inflammation and mortality due to sepsis in a murine model (69). In this study, mice with a zinc-deficient diet had a 50% reduction in plasma zinc levels compared with those with a normal diet, and had a significantly lower survival rate of 10% in the context of sepsis. Based on the studies mentioned above, one could hypothesize that an initial chelation of zinc would trigger an antiviral response mediated by interferon type 1 (IFN-I). However, ensuring an adequate level of zinc would be necessary to regulate this response, since zinc participates as an inhibitory agent at many points in this pathway (see Figure 1). Indeed, an early IFN-I response was shown to be optimal, while a delayed IFN-I response was associated with ARDS in a study with SARS-CoV-infected mice (70). IFN-1 subtypes were studied alone and in combination with other antiviral drugs for the treatment of SARS and MERS, *in vitro* and *in vivo*, with some beneficial reports, but later failed to improve outcomes in humans (71–73). Despite this, SARS-CoV-2 appears to be more sensitive than MERS or SARS-CoV to IFN type 1, and its use as prophylaxis or treatment is also being studied (74). Although it is also hypothesized that it should be tested on the early phase of infection, late phase anti-IFN type 1 treatment could be beneficial for treating severe disease (75). There is some evidence that SARS-CoV-2 infection triggers expression of numerous IFN-stimulated genes, which is thought to induce inadequate IFN responses (76). Although there are no specific data regarding zinc in this pathway for SARS-CoV-2, zinc may limit infection through upregulation of IFN-alpha production and an increase in its antiviral activity (77, 78). In this latter *in vitro* study, when cultures of white blood cells from elderly subjects were supplemented with 15 μM zinc (the physiological concentration), they produced IFN in amounts comparable to those from the younger subjects. We hypothesize that transient zinc deficiency during infection could result in a hyperinflammatory state in those with prior zinc deficiency. Also, zinc deficiency has been linked to a loss of taste and smell, symptoms recently attributed to infection by this virus (79, 80). In our opinion, this could be a consequence of a transient acute zinc deficiency produced during infection. Zinc deficiency may diminish protein synthesis in taste bud cells, reduce alkaline phosphatase activity in taste buds, alter a zinc-containing salivary protein, block the taste pore region of the taste bud or lead to central nervous system dysfunction (81).

IL-6 appears to be important in triggering severe lung damage during SARS-CoV-2 infection. Sustained elevation of IL-6 is postulated as being responsible for severe immune-mediated lung damage as well as for macrophage activation syndrome (MAS) that might overlap in patients with severe COVID-19 (82). There is much evidence for how this cytokine storm may be related to zinc levels. Firstly, IL-6 induces expression of metallothioneins (MT) and alpha-2-macroglobulin (A2M) (both zinc-binding proteins), which can reduce zinc bioavailability. IL-6, MT, and A2M increase with age and impaired zinc availability contributes to immunosenescence (83). Secondly, zinc acts as an anti-inflammatory element, downregulating many pro-inflammatory signaling pathways, such as IL-6-mediated activation of STAT-3 (84). Thirdly, IL-6 production seems to be increased in zinc-deficient elderly subjects. Furthermore, obese patients with lower dietary intake of zinc present with lower plasma and intracellular zinc levels, along with upregulated gene expression of IL-1 alpha, IL-1 beta, and IL-6, compared with patients with higher zinc intake (85). In this *in vivo* study, 10 mg of pure zinc supplementation resulted in a significant 96.5% decrease in IL-6 release from white blood cells in healthy elderly subjects. Fourthly, a polymorphism has been described in the IL-6 gene that is related to impaired zinc homeostasis. An IL-6 promoter gene single nucleotide polymorphism (SNP) at position −174 has been studied in several age-related diseases, such as cardiovascular disease, Alzheimer's disease, diabetes, and cancer (86–88). Zinc deficiency induces a progressive demethylation of the IL-6 promoter in THP1 cells, which correlated to increased IL-6 expression (89). Genetic variation at the IL-6-174G/C locus is involved in determining IL-6 production and the immune response. Elderly subjects with GG genotypes (called C-) have more risk of developing atherosclerosis due to higher IL-6 production, impaired K cell cytotoxicity, increased MT gene expression, and low zinc ion availability compared with C+ carriers (90). For instance, in elderly individuals aged 65–85 years, C+ polymorphism was associated with IL-6 levels of 0.88 pg/ml and zinc levels of 82.2 μg/dl, whereas C- polymorphism was associated with IL-6 levels of 1.21 pg/ml and plasma zinc of 77.5 μg/dl, these differences being statistically significant. In another study, C+ carriers had significantly higher plasma zinc levels, lower MT production, higher red blood cell zinc levels, and good NK cell cytotoxicity, as shown in an *in vivo* study performed in elderly subjects (91). Thus, patients with IL-6-174 GG polymorphism (C- carriers) may be susceptible to developing a severe infection due to SARS-CoV-2, leading to an increase in IL-6 levels that produce a cytokine storm related to impaired zinc homeostasis. Interestingly, this polymorphism seems to be twice as common in people from Italy (68.1%) and other Mediterranean countries, compared with northern European countries such as Germany (33.8%) (91). This might explain, to some degree, the difference in mortality rates observed between these countries; as of the 21 March, Italy recorded 53,578 confirmed cases and 4,825 deaths, while Germany had 22,213 cases and 84 deaths (92). To date, Germany has one of the lowest case fatality rates at 4.10% as of the beginning of May, compared with Italy (13.61%). It is probable that other factors, such as differences in early identification of cases and correct isolation, and differences in the proportion of the population that is elderly, may also have been important. Nevertheless, studies on genetic susceptibility for developing COVID-19 pneumonia and severe illness are underway (93, 94). There are no data regarding the prevalence of this polymorphism in other countries such as the UK or USA, which are known foci of the pandemic. The USA has almost 1,500,000 infected cases with more than 86,000 deaths, which translates to a fatality rate of 5.7% (92).

Zinc has shown its ability to inhibit SAR-CoV RNA polymerase (95). Zn^2+^ cations, especially in combination with Zn ionophore pyrithione, inhibited SARS-CoV RNA-dependent RNA polymerase, RdRP. A more than 50% reduction in overall RNA synthesis was observed at zinc levels of 50 μM, while <5% activity remained at zinc levels of 500 μM. This finding would make zinc a potential antiviral agent for coronavirus diseases. Additionally, chloroquine and hydroxychloroquine, among their other specific mechanisms, act as zinc ionophores and promote cellular uptake of zinc—a mechanism which may increase the effectiveness of these compounds in inhibiting the replicative capacity of the virus (96, 97). SARS-CoV-2 and SARS-CoV require angiotensin-converting enzyme 2 (ACE2) for entry into target cells. Zinc exposure reduced recombinant human ACE-2 activity in rat lung (98). ACE-2 is a zinc metallopeptidase that contains a HEXXH motif that functions as the zinc-binding domain at its active site. In this *in vitro* study, in the presence of 100 μM zinc, activity was significantly (*p* < 0.05) decreased in rat lung and rhACE-2 compared with 0 or 10 μM zinc. In the presence of 1,000 μM zinc, activity was further reduced (*p* < 0.05) in all three preparations compared with 0, 10, and 100 μM zinc. Thus, hypothetically, zinc deficiency could facilitate SARS-CoV-2 infection of target cells due to an increase in ACE-2 activity that could facilitate binding with SARS-CoV-2.

In conclusion, the world is facing a pandemic caused by a novel coronavirus, with some countries suffering a higher burden of disease. The infection is known to more severely affect older people with various chronic comorbidities such as obesity, hypertension, and diabetes. Zinc has a known role in the regulation of immunity. A plausible biological mechanism for the involvement of zinc in this condition exists, which we summarize in Figure 1. Its supplementation, alone or as an adjuvant to medicines that are currently being used to treat active infection, could be beneficial due to its effect on many key factors in the regulation of a severe immune response during infection. Zinc supplementation could be a novel treatment for people at high risk of zinc deficiency who develop severe pneumonia due to Covid-19. We believe there is enough evidence to further investigate how zinc status or homeostasis is involved in the pathogenesis of severe illness produced by SARS-CoV-2 infection, and its potential role as an active treatment should be assessed in clinical trials.

## Author Contributions

All authors listed have made a substantial, direct and intellectual contribution to the work, and approved it for publication.

## Conflict of Interest

The authors declare that the research was conducted in the absence of any commercial or financial relationships that could be construed as a potential conflict of interest.
